# Referral to and engagement in substance use disorder treatment within opioid intervention courts in New York: a qualitative study of implementation barriers and facilitators

**DOI:** 10.1186/s13011-024-00593-y

**Published:** 2024-01-29

**Authors:** Megan A. O’Grady, Katherine S. Elkington, Gail Robson, Ikenna Y. Achebe, Arthur Robin Williams, Alwyn T. Cohall, Renee Cohall, Monica Christofferson, Alejandra Garcia, Kelly S. Ramsey, Pat Lincourt, Susan Tross

**Affiliations:** 1 Department of Public Health Sciences, University of Connecticut School of Medicine, 263 Farmington Ave, Farmington, CT 06030 USA; 2 HIV Center for Clinical and Behavioral Studies, New York State Psychiatric Institute, Columbia University, New York, NY USA; 3 Division on Substance Use Disorders, New York State Psychiatric Institute, New York, NY USA; 4 Center for Behavioral Health and Youth Justice, Columbia University, New York Psychiatric Institute, New York, NY USA; 5 Callen-Lorde Community Health Center, New York, NY USA; 6 Department of Sociomedical Sciences, Mailman School of Public Health, Columbia University, New York, NY USA; 7 Center for Justice Innovation, New York, NY USA; 8 New York State Office of Addiction Services and Supports, Albany, NY USA

**Keywords:** Opioid use disorder, Opioid intervention court, Medications for opioid use disorder, Criminal justice, Opioid overdose, Courts, Substance use disorder treatment

## Abstract

**Background:**

People with opioid use disorder (OUD) are frequently in contact with the court system and have markedly higher rates of fatal opioid overdose. Opioid intervention courts (OIC) were developed to address increasing rates of opioid overdose among court defendants by engaging court staff in identification of treatment need and referral for opioid-related services and building collaborations between the court and OUD treatment systems. The study goal was to understand implementation barriers and facilitators in referring and engaging OIC clients in OUD treatment.

**Methods:**

Semi-structured interviews were conducted with OIC stakeholders (*n* = 46) in 10 New York counties in the United States, including court coordinators, court case managers, and substance use disorder treatment clinic counselors, administrators, and peers. Interviews were recorded and transcribed and thematic analysis was conducted, guided by the Exploration, Preparation, Implementation, Sustainment (EPIS) framework, employing both inductive and deductive coding.

**Results:**

Results were conceptualized using EPIS inner (i.e., courts) and outer (i.e., OUD treatment providers) implementation contexts and bridging factors that impacted referral and engagement to OUD treatment from the OIC. Inner factors that facilitated OIC implementation included OIC philosophy (e.g., non-punitive, access-oriented), court organizational structure (e.g., strong court staff connectedness), and OIC court staff and client characteristics (e.g., positive medications for OUD [MOUD] attitudes). The latter two also served as barriers (e.g., lack of formalized procedures; stigma toward MOUD). Two outer context entities impacted OIC implementation as both barriers and facilitators: substance use disorder treatment programs (e.g., attitudes toward the OIC and MOUD; operational characteristics) and community environments (e.g., attitudes toward the opioid epidemic). The COVID-19 pandemic and bail reform were macro-outer context factors that negatively impacted OIC implementation. Facilitating bridging factors included staffing practices that bridged court and treatment systems (e.g., peers); barriers included communication and cultural differences between systems (e.g., differing expectations about OIC client success).

**Conclusions:**

This study identified key barriers and facilitators that OICs may consider as this model expands in the United States. Referral to and engagement in OUD treatment within the OIC context requires ongoing efforts to bridge the treatment and court systems, and reduce stigma around MOUD.

## Background

In the US, criminal courts are potentially underutilized settings for establishing opioid treatment and overdose prevention initiatives. People with opioid misuse and opioid use disorder (OUD) are frequently in contact with the criminal justice system and have markedly higher rates of fatal opioid overdose and acute healthcare visits than the general population [1–3]. While more ‘upstream’ programs (e.g., diversion to treatment at arrest) are essential, and arguably preferable, many individuals at risk for opioid overdose appear in court untreated [1]. Public defenders, judges, and managers of court-based programs could thus play an important role in identifying those at risk for overdose and connecting them with appropriate services. Opioid intervention courts (OIC) were developed as a novel pre-plea (i.e., at or before arraignment before a formal plea of guilty or not guilty is entered) court model to address increasing rates of opioid overdose among court defendants. This is accomplished by engaging court staff in identification of problem use and treatment need and referral for opioid-related services as well as building collaborations between the court and OUD treatment systems to enhance this cross-systems linkage [4].

The OUD Cascade of Care framework illustrates key stages in addressing OUD, including identification of OUD, treatment, and recovery [5]. The treatment stage consists of several steps including engagement in care, medication for opioid use disorder (MOUD) initiation, retention, and remission. Nonetheless, engagement in care and MOUD initiation is sub-optimal across populations, with studies estimating that as many as 57% of individuals in need of treatment in the US were not engaged in any care and 87% were not receiving MOUD [6, 7]. Even when engaged in OUD treatment, individuals referred by the criminal justice system are substantially less likely to receive MOUD (i.e., methadone or buprenorphine) as compared to those in OUD treatment not referred from a criminal justice source (5% vs. 41% respectively) [8].

In the general population, limited engagement in community-based treatment and MOUD can stem from myriad barriers. These include negative perceptions and internalized stigma about OUD treatment, lack of person-centered, evidence-based care, stigma from family, friends and healthcare providers, logistical issues (e.g., long wait lists, limited available providers), costs, lack of insurance, lack of flexibility in treatment program regulations, and social factors that limit the ability to engage in treatment (e.g., transportation, housing status) [9, 10]. Individuals involved in the court system who are being linked to community-based treatment may face additional barriers, such as challenges in interorganizational relationships between court and substance use disorder (SUD) treatment providers (e.g., low degree of trust in local SUD treatment providers among court staff) and a lack of providers near the court [11, 12].

Additionally, although MOUD is considered the standard of care for OUD, access has historically been limited in problem solving courts like drug courts, especially for agonist-based modalities [8, 13]. While progress has been made in increasing MOUD access within drug courts [14], previous research on drug courts that may be relevant to new court models, like OICs, also show that barriers to MOUD access for drug court clients occur at multiple levels. These may be institutional (e.g., policies restricting use of MOUD), programmatic (e.g., abstinence-only orientation), attitudinal (e.g., negative attitudes), and/or systemic (e.g., linking people from the court system to the treatment system) [15–19]. However, OICs have the potential to address the treatment shortfalls across several of the OUD Cascade of Care steps by addressing these known barriers and more rapidly linking court-involved individuals to treatment in the community. For example, they were designed to be rapid response programs to stabilize individuals at risk for overdose using immediate screening and linkage to treatment programs, initiation of MOUD, intensive judicial monitoring, and recovery support services [4]. OICs aim to reduce overdose, untreated OUD, and recidivism. Therefore, by design these programs address many of the logistical issues, social factors, stigma and negative attitudes, and institutional policies described above that have limited engagement in community-based treatment, and especially MOUD, for those involved in the court system.

The country’s first OIC opened in Buffalo, New York in 2017 and since that time the New York State (NYS) Unified Court System has expanded the model throughout each of the State’s judicial districts. The OIC model is innovative because, in addition to emphasizing rapid screening and linkage to community-based treatment providers (i.e., within 24 h), it is *pre-plea*. Thus, involvement in the OIC happens before a plea (i.e., guilty or not guilty) is entered and prosecution of the case is temporarily paused so that the legal process does not interfere with rapid engagement in treatment and stabilization. Ideally, to minimize coercion, the OIC is set up so that participation has no bearing on the outcome of a pending charge, but it must be acknowledged that the participant has been arrested and awaits potential prosecution. This differs from the typical drug court model in that most are *post-plea*, such that a defendant pleads guilty to charges and opts to participate in a drug court program as a dispositional sentence [20].

Recent work examining outcomes in the first OIC in Buffalo, NY found that OIC clients received treatment sooner and more frequently than a comparison group of defendants who used opioids and were arrested and booked into jail before the OIC began. Further, OIC clients on MOUD were less likely to die in the twelve months after jail booking than those in the comparison group that also had received MOUD [21]. In addition, clients who received MOUD during their time in the OIC had greater odds of completing the OIC compared to those who did not receive MOUD, especially if they received MOUD within the first seven days [20]. Qualitative interviews identified barriers to defendants’ participation in the OIC, including not having reliable transportation, limited social supports, struggling with requirements of the OIC (e.g., daily court appearances), limited motivation to participate, and long-term sustainment of MOUD after OIC program completion [20].

The OIC model is early in implementation in the US. As far as we are aware, the research that currently exists on the model is limited to the first program in Buffalo, NY. As noted above, the OUD Cascade of Care identifies a critical, yet poorly addressed, step in care as engagement and initiation into treatment. Little is known about implementation barriers and facilitators or important contextual factors that may affect how the OIC moves people through this critical early part of the Cascade. These barriers and facilitators to engagement and initiation in treatment among OIC clients may differ substantially from those in the general population as well as those in drug courts. For example, the OIC model by design requires collaboration between justice and treatment systems to achieve rapid and successful cross-systems referral, linkage and treatment initiation. However, such cross-system collaboration often can be hindered by conflicting missions and different norms and attitudes regarding OUD treatment, especially MOUD [22]. Further, the OICs are meant to provide very rapid linkages to treatment and reduce barriers to access linked to social issues (e.g., transportation, stigma, institutional policies, and inter-agency collaboration). Research is needed to understand whether the OICs can address these significant barriers to engagement and initiation in treatment.

The goal of the current study is to address the limited understanding of implementation barriers and facilitators regarding two critical points of the OUD Cascade of Care: referral to and engagement in (i.e., initiation) SUD treatment in the context of the OIC model. In OICs, referral and linkage to treatment is a multi-pronged process that starts with identification/case finding of potential new OIC participants and then screening and assessment for eligibility. Eligibility is informed by guidance from an OIC working group [4]. Once individuals are enrolled in the OIC, linkage to a treatment program occurs, with the goal that linkage is rapid. MOUD is emphasized in referral/linkage and individuals can be referred to any level of care (e.g., inpatient, outpatient) that is appropriate and available. Staff involved in the identification and linkage process (e.g., peers, court coordinators, case managers) as well as processes for identification, screening, and referral vary by county. To systematically explore the facilitators and barriers at each of these OUD Cascade of Care steps, we use the Exploration, Preparation, Implementation, Sustainment (EPIS) framework [23]. The EPIS framework identifies necessary structures and processes within systems to support implementation of evidence-based practice [24] across phases of implementation. Importantly, the framework allows for exploration of barriers to implementation and sustainability because it considers the multilevel nature of service systems. It addresses *outer contexts* (e.g., SUD treatment program characteristics, service availability, and staff attitudes; community attitudes towards opioid overdoses) and *inner contexts* (e.g., OIC organizational policy/philosophy, structure and operations, and staff and client characteristics). It also addresses those key elements that serve a bridging function between contexts (e.g., inter-relationships and coordination between OIC and SUD programs; staffing models that bridge OIC and SUD treatment systems). Given the EPIS approach to exploring the full system in which an intervention (i.e., the OIC) sits in order to understand the key drivers that influence the implementation process, it has been useful in examining cross-systems work in other studies [25, 26]. Therefore, framed by the EPIS framework, this paper qualitatively explores inner and outer contexts and bridging factors to identify facilitators and barriers to referral, linkage and treatment initiation. Semi-structured interviews with county OIC staff (coordinators and case managers) and local community treatment providers during the implementation phase of OICs in 10 counties in NYS were conducted. Results may be useful in identifying implementation strategies to address barriers and facilitators to OIC implementation as the model continues to scale up in the US.

## Methods

### Participants and sampling

Participants were recruited consecutively for semi-structured interviews between 10/2020 and 04/2021 as part of a broader study to explore facilitators and barriers to OIC development and delivery within 10 NYS counties [27]. Interviews took place following the elimination of cash bail in NYS, following the initial wave and statewide shut down due to the COVID-19 pandemic. This was also before a change in policy mandating MOUD availability in NYS jails. Study participants were recruited for interviews if they were identified as stakeholders in their OIC and were involved directly in referral, linking, and/or treatment activities of opioid court clients. OIC stakeholders typically include those who have a relevant role in the support of the OIC in their county, for example presiding judge, court coordinator, county defense attorney, treatment providers, or peers; stakeholder group membership may vary from county to county. Study participants (total *n* = 46) were court coordinators (*n* = 10) and case managers (*n* = 8) drawn from opioid courts, and community SUD treatment providers, supervisors or clinic administrators/directors (*n* = 19) and peers (*n* = 9) from partnering treatment agencies in 10 counties in New York State; 71.7% were female; 65.2% were white, and 2.2% were Hispanic/Latinx. All study procedures were approved by the New York State Psychiatric Institute Institutional Review Board.

### Procedures

Participants were informed of the study and invited to participate via email. Participants then were sent an information sheet before the interview about the project. This sheet was reviewed by a study team member on the call prior to the interview, after which the interviewer took verbal consent to participate in the research. Interviews were conducted via Zoom by one of ten trained interviewers with either a Juris Doctor or a Master’s degree in psychology, social work, or a related field. All interviews were audio recorded to facilitate transcription and took approximately 45 min; due to court and clinic rules around renumeration, participants did not receive compensation for their time.

Interviewers conducted semi-structured interviews following an interview guide developed to inform technical assistance procedures for the OIC. The interview guide asked about facilitators and barriers to implementing OICs as well as communication and relationships with other OIC stakeholders. These interviews included questions examining the following domains: knowledge of and attitudes toward the OIC, activities that occur within the relevant OUD Cascade of Care steps: screening and identification, referral, treatment linkage and MOUD initiation; engagement of court clients in court/treatment; availability of treatment services/programs; attitudes towards and knowledge of MOUD; interagency collaboration; community programming to support recovery.

### Data coding and analysis

All transcripts were de-identified and entered into *NVivo*, a qualitative data software package. We conducted a thematic analysis of the interview texts, employing inductive and EPIS-driven deductive coding approaches. The initial coding scheme focused on identifying facilitators and barriers at each stage of the OUD Cascade of Care (including referral to OICs, OIC screening, referral from OICs to treatment, treatment linkage, MOUD initiation and retention) as well as practices and needs of the OIC (e.g., training needs, linkage gaps) and SUD treatment system (e.g., treatment gaps, continuing care).

The preliminary codebook was developed by six experienced coders, who carried out an initial open coding phase together with three interview transcripts in which narratives pertaining to the above domains were examined and primary codes applied. In the next phase, another three interviews were coded to clarify and expand the description of the primary codes and define secondary codes. Through this process, inter-rater agreement was established, with all coders attaining agreement during the course of consensus discussion, and any initial divergence being resolved through this process. Following coding of these initial interviews, and using the resulting codebook and supporting specifications, each interview was double-coded. In cases where there was disagreement among the raters, this was resolved by discussion. Decision trails were documented to elucidate the systematic procedure through which coding was accomplished, and to assure that interpretations were supported by the data. Only rarely, and if necessary, the codebook was expanded to capture novel responses. This coding process is standard in qualitative methods and known as focused and intensive coding for primary and secondary themes [28, 29].

In *NVivo*, we ran code reports for each step in the OUD Cascade of Care. These reports were the basis for analysis and interpretation of the coded transcripts. Each code report was reviewed by 2–3 researchers to identify EPIS-driven facilitators and barriers across different respondent types relevant to screening, referral, linkage, and treatment initiation, respectively. Thus, EPIS inner setting, outer setting, and bridging factors across systems components were of particular salience. During the analysis and interpretation process, summaries were constructed, of primary and secondary themes, clustering codes within interviews, and discerning patterns of convergence or divergence across respondent types (e.g., court staff and treatment providers) (Dey, 1993). In this paper, we are focused on analysis of referral and linkage to treatment.

## Results

**Fig. 1:**
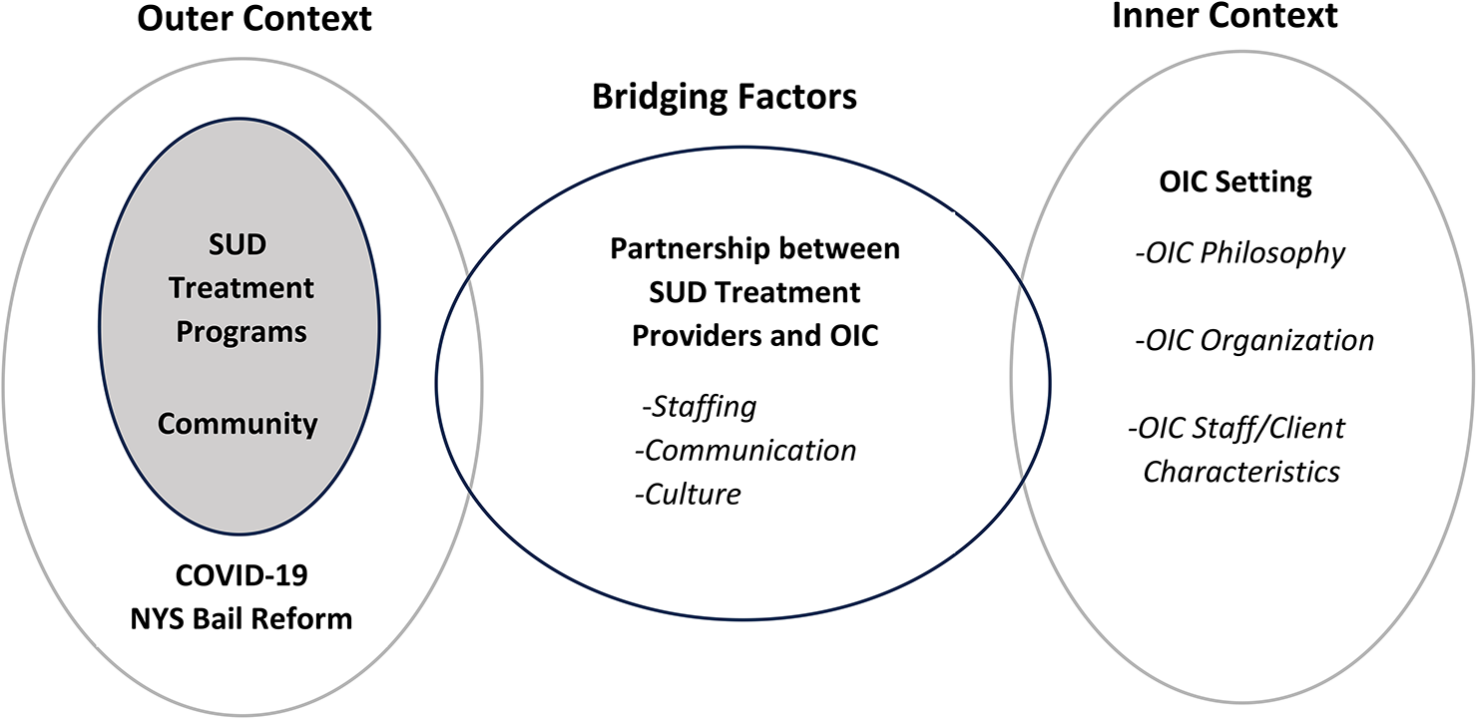
Opioid intervention court EPIS framework constructs. Key: OIC = opioid intervention court; SUD = Substance Use Disorder

The EPIS framework (See Fig. 1) offers a paradigm for conceptualizing the evolving referral and linkage to treatment operations of the OIC, including the inner and outer context and bridging factors. For each EPIS component, constructs acting as facilitators and barriers to the OIC functions were distinguished. Stakeholders of different types– court coordinators, case managers, treatment providers and peers– although speaking from different vantage points, largely expressed the same perceptions of the referral and linkage operations of the OIC. Thus, themes presented pertain to the whole sample, with variations and/or nuances by stakeholder type distinguished where applicable.

### Inner context factors

EPIS Inner Context influences refer to the characteristics within an organization that may influence the implementation of evidence-based practice. For the OIC, EPIS inner context elements or characteristics that influence referral and linkage to treatment consisted of three main themes: (1) OIC policy/philosophy, (2) OIC organizational structure and operations, and (3) OIC court staff and client characteristics. Below, these three themes are discussed separately, and ways in which factors in each function as facilitators, barriers, or both are described.

#### OIC policy/philosophy factors

The first unifying philosophy or policy of the court model was the court’s focus on treatment stabilization of court clients to reduce the risk of overdose, as opposed to substance use or relapse, as a means to reduce and prevent recidivism as seen in traditional drug court models. Respondents appreciated that the overdose-centered approach was born from the court’s recognition of the opioid crisis.

A second unifying philosophy was a person-centered approach to non-mandated treatment whereby court staff work with the court client to develop an initial treatment plan that is acceptable to the client with an eye toward stepping up treatment consistent with a client’s needs over time. As one court coordinator noted, this is a distinct difference from a traditional post-plea treatment court:

*So it works differently than the other problem-solving courts right because this is not ‘you sign a contract, and you have to do it,’ this is ‘we want to meet you where you’re at–this is what I think you need but you’re not willing to do it, so what about this?’. If they say no, ‘what about this?’…we try to work with the person until they agree to commit to something, even if it’s just medically assisted treatment*.

Importantly, not following the recommended treatment plan did not result in dismissal from the court or increased legal sanctions. This approach was notable to providers who observed further investment in the client and a review of additional supports to encourage treatment engagement as opposed to punishment:

*You know, judge would ask– and sometimes say, “You look like shit. What happened last night? How are you feeling?…Do we need to find another resource to help link you with treatment? Do you need additional support?” In that capacity, you could really see that the judge was invested.*

While many narratives of both providers and court staff praised the philosophy of the new court model, some providers noted the model could go even further to be more inclusive of a high-risk polysubstance using population that frequently comes into contact with the legal/court system. In particular, one provider noted that the ever-evolving opioid crisis required the court model to adapt to a polysubstance use approach to deal effectively with court clients at risk for overdose due to polysubstance use or fentanyl contained in non-opioid substances, such as stimulants:

*We’re not dealing with pure substances anymore. We’re dealing with this kind of concoction which includes fentanyl, you know?…So we’re seeing, like, latency of this kind of opioid use disorder when really, you know, we’re dealing with a stimulant use disorder.*

#### OIC organizational structure

Court philosophy and policy strongly informed a court organizational structure designed to improve referral and treatment linkage. In particular, the connectedness of OIC staff (e.g., court coordinators, case managers) with other operations/parts of the courthouse (e.g., arraignment) or closely connected facilities (e.g., jails) to facilitate identification of potential court clients emerged as a critical way to eventually engage clients in SUD treatment. For example, jails are a frequent touch point for OIC clients while they are interacting with the courts and coordination with jails is sometimes required to engage OIC clients in treatment. However, there were barriers to coordination with jails to facilitate treatment linkage and some participants indicated this may be due to jail systems’ ambivalent or negative stance toward MOUD. For example, a court peer indicated that jails present bureaucratic challenges for treatment linkage, around release and escort to treatment. On the other hand, it was observed that jail attitudes toward connecting people to MOUD was changing gradually, and that jails in some counties were shifting to become an active source for engagement with the OICs to engage clients in treatment.

Nonetheless, organizational characteristics also emerged from participants’ narratives as barriers to OIC function and client participation in the court. This was attributed to the newness of the court model in the counties included in the study. Notably, a lack of formal policy and procedures for some courts was noted as a barrier to successful court operation, for example being able to communicate specific inclusion or enrollment criteria to referring parties or for court completion. In addition, SUD treatment providers perceived court screening or assessment of opioid use issues as insufficient. Recognizing that court staff may not be trained to make clinical determinations, providers described logistical barriers to accessing court clients in a timely fashion to conduct an assessment, and ensure the person was appropriate for the court:

*…what we’re kind of lacking is a bigger assessment piece that we need on our side just to make sure it’s an appropriate fit…I know the court’s trying to expedite, you know, just by filling out what they did. But as we’re going through this process, we’re realizing it’s really not enough.*

#### OIC staff and client characteristics

In addition to policy/philosophy and organizational characteristics, themes related to staff and client characteristics emerged as important in the success of the OIC model. In particular, beliefs and attitudes towards MOUD held by court staff and clients served as both facilitators and barriers to OIC success. Providers noted a strength of the OIC model is the court’s focus on MOUD, which has translated into advocacy on the part of court staff for court clients’ access to it. As one provider notes, “*I think that opiate court is definitely an advocate for it…I think they definitely believe in its worth*.”

However, the support and advocacy for MOUD was not a universally held tenet across court staff with providers noting that court staff may follow the model with respect to MOUD while still maintaining stigmatizing or non-favorable attitudes toward MOUD, “I’ve just gotten the feel from other members that maybe not everyone else is kind of on the same page. I think they follow the recommendations but…I’m not sure if everyone is on board about MAT.”

Study participants perceived that OIC clients’ motivation to engage in treatment, or ambivalence thereof, may impact the success of the OIC model. In contrast, due to the non-punitive nature of the OIC, some court staff described a commitment to addressing court client ambivalence around treatment as tenuous and diminishing clients’ ability to engage and be successful in the court. Nonetheless, the person-centered nature of the court model allows for, as noted by a case manager, *“a very long process of courts meeting them [clients] where they’re at and engaging them in the treatment that they are willing to do”* to achieve success by addressing motivation over the longer term.

### Outer context factors

In the current study, treatment programs served as the primary outer context factor in OIC implementation; the community environment served as an additional factor. Ways in which these factors served as facilitators and/or barriers are distinguished, including when the same influence served as both.

#### SUD treatment programs

SUD treatment programs are crucial partners in OIC referral and linkage operations. Several treatment program characteristics, attitudinal and/or structural factors emerged as salient influences on these operations. These fell within the domains of (1) treatment program attitudes toward the OIC and MOUD and (2) operational characteristics.

#### SUD treatment program attitudes toward the OIC and MOUD

Positive experiences with and perceptions of the effectiveness of the OIC were commonly expressed by SUD treatment programs who collaborated with the OICs. The OIC was characterized as a powerful catalyst for client connection to MOUD as a frontline approach to OUD treatment, attainment of substance use goals, and comprehensive client recovery. Treatment program perception of the OIC, like other intervention courts, as pathways for multi-faceted, enduring personal change was cited. In particular, treatment programs perceived the OIC as uniquely positioned to achieve this impact more so than other intervention or problem-solving courts. As one provider stated:

*We have seen the successful completion and the reduction in charges for several individuals…watching the growth of those individuals who remain, to this day, some of the first participants who went through still sober which, to me, is just remarkable because they reach back out to us frequently to say, like, “I’ve tried many times. Nothing else has ever worked”.*

Additionally, as with court staff, though to a lesser extent, negative attitudes towards MOUD were mentioned among a subgroup of providers, themselves members of the recovery community, that served as barriers to collaboration with the more MOUD-positive OIC.

### Treatment program operational characteristics

Procedures and resources for working with OIC clients, were cited commonly as major factors positively influencing OIC referral and linkage to treatment programs. When treatment programs were numerous in the region or had explicitly dedicated resources, procedures and/or staffing for OIC clients these served as significant facilitators to referral and linkage. This was observed by OIC coordinators, case managers, peers, and SUD providers alike. For example, providers described the crucial benefit of rapid same-day buprenorphine intake and initiation procedures for OIC clients. Further, some SUD programs had dedicated and prioritized intake and treatment slots specifically for OIC clients. As noted by an OIC case manager, “*they’ve (SUD treatment provider) made available during certain days and certain times– every single day appointments for individuals to immediately get appointments if they’re being referred from opioid court*.” Providers confirmed this operational policy noting that they were able to see OIC clients same day or within 24 h at a maximum.

However, when there are limited, strained, or no OIC-dedicated resources, funding, procedures, and staffing, these SUD program characteristics pose barriers to OIC referral and linkage to treatment. Specifically, lack of services on weekends, lack of certain services (e.g., methadone treatment, dual diagnosis services, and inpatient facilities), and staff turnover were noted.

#### Community environment

Community sensibility and attitude toward the opioid crisis created a community environment which was both facilitator of and barrier to OIC operations. In those communities where perception of opioid use as a public crisis was held, community environment was a significant facilitator of OIC operations. As one SUD treatment provider observed, due to this sense of urgency, collaborative structures were formed, which could be forces for change in policy and practices, “*We’ve had an opiate taskforce. And we recently decided that we were going to be more of a community-wide opiate and other substances taskforce.*” However, other communities were characterized as poorly informed and indifferent. As one SUD treatment provider stated, “*There are a lot of barriers and not just on the court side or law enforcement. It’s just in general. The community doesn’t want to admit there’s a problem.*” In addition, ongoing stigmatization of people who use opioids, as well as other substances, was a potent barrier to OIC operations and failure to refer to the court and/or to other services. As one SUD provider stated:

*We certainly do see the stigma when it comes to MAT…we’ve made some strides there as far as educating the community on how these medications benefit individuals and can help them get their lives back on track.*

In part, this was attributed to community providers– including primary care providers, other medical providers, and mental health providers, and especially those in the recovery community, who could, otherwise, be frontline sources of referral.

### Macro-level outer context

#### COVID-19 pandemic public health environment

The COVID-19 pandemic public health environment, defined by a full range of mitigation or restrictive strategies for infection control, served as a potent barrier, at outset, and as both barrier and facilitator, later, to OIC operations. These strategies consisted of shelter-in-place lockdown at the outset of the pandemic, requirement for social distance and masking (i.e., at first, mandated, and, later, strongly encouraged) in shared environments, testing prior to entry into shared environments, and vaccination. NYS courts were closed for the first months of lockdown and all operations were halted. As operations started up again, the OIC was markedly interrupted. As one court case manager observed, “*During COVID, we really had hardly any referrals– maybe only a couple people coming through.*” However, as mode of OIC operations pivoted to telehealth, for those who were technology-equipped and proficient, conduct of procedures and delivery of services through virtual platforms served to ease linkage to treatment. At the same time, for those individuals who were not, use of these platforms posed an obstacle. As one court case manager observed, “*During COVID, it was really difficult to schedule clients. A lot of them didn’t have access to remote technology.*” Further, these COVID-19 pandemic-related systems transformation, as well as COVID-19 related staff losses, due to illness and/or turnover, imposed added strain on remaining staff.

#### Non-cash bail reform

The criminal justice legal environment is a potent outer context factor impacting OIC operations. Within this environment, recent reform in bail policy [30], significantly loosening bail requirements for people entering the courts, has served as a barrier to OIC referral and enrollment. Bail reform was described by some participants as removing individuals’ incentives to participating in OIC. As one provider observed, it “*killed the hook for [Opioid Court participation] because the hook was to keep them out of jail and get them treatment.”* Due to this, participants attributed decreased volume of clients to bail reform. In addition to this policy change, a second practice change in the criminal justice legal environment was cited as an important barrier to OIC operations. This was increased lag time between arrest and arraignment (i.e., the latter of which would have been the time point for OIC intervention). As one court case manager reported, *“Even though the goal is to get them assessed within twenty-four hours of being arraigned, often they’re not arraigned right now for seven months after they’re originally arrested, or longer.”*

### Bridging factors

The bridging factors component of EPIS recognizes the interconnectedness and relationship between outer and inner context entities. In the case of the OICs, clients and staff must navigate from the inner setting of the court system and OICs to the outer setting of the SUD treatment system. Better interconnection between these settings can result in rapid linkage from the OIC to SUD treatment and potentially improvement of outcomes for OIC clients. Below we review factors that facilitated the bridge between inner and outer settings and factors that were barriers to this bridging, including staffing practices, communication between systems, and cultural differences.

#### Staffing to bridge systems

Staffing models played a role in facilitating OIC implementation. For example, the practice of having SUD treatment staff housed within the court, bridging the gap between systems, was noted as an implementation facilitator. As one provider noted:

*Having a co-located substance use counselor in the court has been beneficial in a lot of ways. So, we have a treatment liaison on the court team which is nice, but to have someone really embedded in the court, I think, has been really useful for the court staff specifically.*

The use of peers also served to enhance client connection between the court and treatment systems due to both relational and logistical/practical support the peer can provide inside and outside the courtroom setting. A peer described the unique strengths of their role and approach in achieving court client engagement over repeated and frequent contact that can facilitate the client’s connection to services:

*…I can kinda pull them right in, I can pull strings and get them appointments quicker and I think even just when I pull someone in from working with them, like I call them every day…and it just opens their eyes to realize that they’re not alone…*

#### Communication and culture

However, there were barriers that impeded coordination between court and treatment systems. For example, communication between staff from treatment programs and OIC programs about treatment assessment, progress, and completion was either limited or inconsistent. As noted by a treatment provider about the OICs, “*We don’t particularly get much information from them, and I don’t know that -- if that’s because we have been familiar with the patient within the system at this point or if we do -- I’m not sure why.”* Thus, creating communication procedures to share assessment information between the OIC and SUD treatment providers would help set the stage for coordination. Further, clients and staff had to adapt to different cultures within each of the systems that may have different expectations and languages to indicate success and progress. For example, OIC clients encountered the marked shift from criminal justice system involvement to the therapeutic activities of SUD treatment programs. As noted by a provider,

*There’s often, culture difference between the court system and the substances system, and I think it’s been challenging in the past for some of our providers and their peer staff, to adequately prepare individuals who are engaged in court for what comes next, and to be able to prepare them for success*.

## Discussion

Initial evaluations of the first OIC in Buffalo, NY has shown the model to be successful at engaging court populations that may have been previously underserved by the court and SUD treatment systems for OUD and overdose risk [21]. Further, there is evidence of more rapid treatment entry and initiation of MOUD in OICs as compared to drug courts [31]. Findings from this paper extend what is known about OIC implementation, which is critical given the burgeoning interest in the success of the model, both within NYS and across the US. Understanding those elements within the court and the treatment system that serve to support or hinder the success of the OIC model and related outcomes, including reduced overdose, initiation of MOUD and reduced recidivism, is critical in developing implementation strategies that can support the development and scale-up of this court model. Our qualitative approach was a strength given that we were able to gain in depth-understanding of OIC implementation barriers and facilitators from the perspective of multiple stakeholders from both the court and SUD treatment systems.

We found that three main *inner context* factors contributed to implementation of the OICs. These included court philosophy (non-punitive, access-oriented), court organization (strong connectedness among OIC and non-OIC staff within the courts), and staff and client characteristics (pro-MOUD rather than stigmatizing). In terms of court philosophy, participants felt the court focused on stabilization and overdose prevention, providing resources, and access to voluntary treatment consistent with clients’ needs. Mainly, the OIC does not rely on dismissal and legal sanctions. This philosophy represents a clear shift from approaches that the court system and other problem-solving courts have used with people who use drugs. This philosophy also addresses key barriers to accessing MOUD, such as lack of flexibility in regulations, and moves towards a more person-centered orientation [9]. A person-centered care approach in SUD treatment has been shown to improve utilization of evidence-based services and is associated with other positive outcomes (e.g., treatment satisfaction) [32, 33]. Therefore, this person-centered philosophy should be a key feature in the implementation of OICs. Additionally, participants noted concerns related to the shifting nature of the opioid epidemic such that OICs may need to evolve to identify and address polysubstance use. Therefore, screening tools and admission criteria may need to be broadened given that other substances pose overdose risk (e.g., cocaine with fentanyl).

The way the OIC was structured and organized also impacted implementation. It was clear that a number of court staff and officials needed to work together in order to identify appropriate OIC clients and rapidly connect them with the OIC program. Therefore, strong connectedness between various OIC staff and systems is required for program success. Because of the newness of the OIC model, OIC programs sometimes lacked formal policies and procedures. New OIC programs should ensure that formal policies and procedures are in place from the beginning to meet the goals of rapid linkage and treatment for those at risk of overdose. One specific barrier noted was that different tools were being used across OICs to identify and assess individuals for the OIC, some of which study participants felt were insufficient. This issue also has been noted in drug courts. For example, one study found that many drug courts did not appropriately use screening and assessment instruments for placement decisions [34]. Further development of efficient and practical screening tools for OICs may be warranted.

In terms of staff characteristics, participants noted that OIC court staff generally advocated for use of MOUD, which is a shift from the drug court model in which MOUD traditionally has been stigmatized. However, providers and peers also noted that MOUD stigma still exists, suggesting that ongoing training and advocacy for MOUD within court systems must continue as OICs are rolled out. On the court client side, motivation to participate represented both a barrier and a facilitator. Similarly, common reasons for early termination in drug courts include lack of engagement in SUD treatment and low motivation to participate [34]. Providers perceived that some OIC clients were ambivalent about participating in the OIC or engaging in treatment; however, a benefit of the OIC model is that it includes intensive and ongoing outreach and engagement with clients to try to address this ambivalence. Often peers and case managers played a big part in these ongoing engagement attempts. Peers with lived experience who can connect to clients as credible role models and supports are especially appealing to support ongoing engagement. Further, OIC staff should be trained in approaches to address ambivalence, such as motivational interviewing, that emphasize meeting clients where they are at, highlighting an important skill to develop, and study the implementation of, among OIC staff.

Our findings also highlighted the powerful role of two major *Outer Context* entities on OIC function: treatment programs and the community environment. As direct collaborators with the OIC in offering services, SUD treatment programs were an extremely important outer context influence on OIC function. This influence occurred in several ways. First, SUD treatment programs generally had positive attitudes about the OIC, seeing it as a unique contributor to client success. This point was epitomized by an SUD treatment provider who poignantly reflected that the OIC had transformed their clients who had experienced multiple negative impacts from substance use, when all else had had little impact. Second, treatment programs were described as core resources for rapid provision of OUD treatment and supportive services essential for clients’ recovery. This is very similar to the description of the benefits of drug courts [35]. However, for both OIC and drug courts, our findings and those of prior authors warn that this benefit only can occur in the setting of an adequate supply of SUD treatment programs as well as availability of sufficiently broad recovery support services to meet clients’ basic needs [36]. In our findings, as well as those of others describing drug court function, the wide variability in this supply of services is a serious barrier to fulfilling court goals [34, 37]. Third, some SUD treatment programs also were described as potential sources of negative biases, or stigmatizing attitudes about MOUD that served as barriers to OIC function. Rarely, but notably, participants described OIC support for MOUD as a stimulus for reluctance to collaborate with the OIC. This was attributed especially to SUD treatment providers who were ardent members of the self-help recovery community, and who viewed use of MOUD as compromising true (i.e., medication-free) recovery [38]. Therefore, continued emphasis on building treatment system capacity both in terms of density and non-stigmatizing attitudes toward MOUD will be necessary for future success of OICs.

Our second major outer context entity, the community environment, was identified as a sometime facilitator of, and sometime barrier to OIC function. First, awareness of and effort given to alleviate the opioid and overdose epidemics, or the lack of these, was described. Namely, when the OIC and SUD treatment programs were based in a community with a high level of information about the opioid and overdose epidemics as a public health crisis, this culture of shared concern can serve as a facilitator to OIC function. When there is normative acknowledgment of the need for public education and resources to alleviate this crisis, this can enhance activity all along the OUD Cascade of Care, beginning with referral to the OIC and to SUD treatment. However, some of our participants also lamented the dampening or numbing effect of community indifference, wherein the overdose epidemic has continued to be neglected. Second, community stigmatization of people who use drugs and those who use MOUD for their recovery was described as a barrier to OIC function. When there is normative stigmatization and marginalization of people who use drugs, and stigmatization around MOUD, this are powerful impediments to recovery-positive attitudes and actions that are imperative for alleviating the opioid epidemic. Therefore, to support OIC implementation, building non-stigmatizing attitudes among treatment and court providers/staff, as well as engaging local communities by emphasizing the importance of addressing the opioid epidemic and using evidence-based, person-centered practices, should be ongoing.

Two macro-level outer context factors were noted as important in our results: bail reform and the COVID-19 pandemic. One of several factors which can affect the implementation, expansion, and ultimately the success of the OIC model, is bail reform. Briefly, many municipalities began assessing fees and fines to those arrested for low level offenses or as an alternative to incarceration strategy for minor crimes, such as misdemeanors. However, many individuals lack the means to post bail and pay fees/fines. For example, at any given time it is estimated that one-third of incarcerated individuals are in jail, pre-trial without yet being convicted of a crime [39]. Recognizing disproportionate impact of levying fines, fees and bail payment on poor people and people of color, many states, including NYS, have developed strategies designed to provide more equitable results and reduce inappropriate confinement for those individuals who have committed low level offenses and who lack the resources to pay fines, fees or post bail [30, 40]. For example, in NYS, individuals receive a desk appearance ticket as the modality of citation for offenses that are no longer eligible for jail. Bail reform has resulted in lower incarceration rates for individuals arrested for minor offenses. Thus, for many individuals the appeal of going to a treatment court to avoid sentencing is minimized. In some NYS jurisdictions, the number of people enrolled in OIC programs has dropped substantially. For example, in Rochester NY, there were 60 people enrolled over a two-month period in 2019 as compared to 7 people in 2020 after bail reform had taken effect [41]. Thus, bail reform policies could potentially pose a longer-term issue with engaging individuals in OICs. Policy, legal, and OIC operational strategies to overcome these unintended consequences should be investigated and tested. Further, one of the OICs original intensions was to enroll individuals with broad legal eligibility [4], including those with more serious charges, and not just those with minor offenses; thus, the characteristics of those enrolled in OICs should be examined.

Another macro-level outer context factor affecting implementation and utilization of OICs is the COVID-19 pandemic. Incarcerated individuals, particularly those with OUD, are at higher risk both for acquisition as well as succumbing to the SARS-CoV-2 virus [42]. Recognizing this vulnerability, and the inherent risk to staff and personnel, prisons, jails and law enforcement personnel enacted several strategies to mitigate risk, including decarceration of inmates with low level offenses and reducing arrests at the community level [43]. Both have implications for OICs. With respect to the former, release of incarcerated individuals often occurred without the ability to secure adequate linkages for MOUD care in community settings. This was exacerbated by limited capacity of community providers to accept new clients due to their own restrictions imposed by the COVID-19 pandemic. Further, reduction in arrests led to decreased opportunities for individuals to appear in OIC and avail themselves of whatever available treatment services were still operational.

Finally, bridging factors were barriers and facilitators related to inter-relationships and coordination between treatment programs and the OICs. For example, embedding treatment staff on-site in the OIC and using peers to help bridge the court and SUD treatment systems had a particularly facilitative impact. However, communication between SUD treatment staff and OICs and differences in culture and expectations between the systems were noted as a barriers. These findings suggest that as OICs are starting and continuing their operations, building inter-relationships and alignment via shared discussions among stakeholders from the treatment and court systems on general operations (e.g., staffing and communications) as well as expectations and potential cultural differences between the systems will be necessary.

While this study has many strengths, there are a few limitations to note. First, this study focuses only on treatment and court staff and not on judges and lawyers working within OICs; while their perspectives are important, for the purposes of this study, we interviewed those most directly working within the OICs on treatment referral and initiation. Further, we did not interview OIC clients or individuals in OUD treatment; future research should examine OIC court clients’ experiences from an implementation lens. Second, this study was limited to ten counties in NYS and may not be representative of the entire state or other states. Finally, as noted above, this study was conducted during the early part of the COVID-19 pandemic and as bail reform was occurring; therefore, some results may be influenced by these macro-contextual factors.

## Conclusions

This study provides important information about barriers and facilitators in the implementation of OICs, a court model that is relatively new and growing in the US. Yet, the policy and practice environment in which OICs may be implemented continues to evolve. For example, there are two recent developments that may help improve *Outer Context* entities bearing on OIC functioning related to treatment programs and the community environment. Foremost are recent court cases leading to billions of dollars in opioid settlement funds that will be available to states and communities to scale access to affordable treatment services. To the extent OIC clients faced limited treatment program availability, settlement funds could add to the current array of options, including telehealth for patients in more remote locales. Additionally, there has been growing awareness of the importance of connecting justice-involved individuals with MOUD. A growing number of states are allowing for MOUD, not just in jails, but prisons as well. For example, NYS now mandates the offering of all forms of MOUD in correctional facilities as well as strategies for connecting inmates with MOUD upon community re-entry. It is unclear how greater availability and acceptance of MOUD in carceral settings may influence knowledge and beliefs of others working outside of institutional settings in the criminal justice universe. However, such policies may serve as a facilitator for OIC implementation both in terms of improving attitudes related to MOUD as well as encouraging coordination between correctional facilities and community treatment providers. Therefore, continued study of the OIC model will be imperative moving forward.

## Data Availability

The datasets generated and/or analysed during the current study are not publicly available due to their qualitative nature.

## Abbreviations

MOUD: Medications for Opioid Use Disorder
NYS: New York State
OIC: Opioid Intervention Court
OUD: Opioid Use Disorder
SUD: Substance Use Disorder
